# Examining the use of metaphors to understand the experience of community treatment orders for patients and mental health workers

**DOI:** 10.1186/s12888-016-0791-z

**Published:** 2016-03-31

**Authors:** Sharon Lawn, Toni Delany, Mariastella Pulvirenti, Ann Smith, John McMillan

**Affiliations:** Flinders Human Behaviour and Health Research Unit, Department of Psychiatry, Flinders University, Room 4 T306 Margaret Tobin Centre, PO Box 2100, Adelaide, South Australia 5001 Australia; Southgate Institute for Health, Society and Equity, Flinders University, Adelaide, South Australia Australia; Discipline of Public Health, Flinders University, Adelaide, South Australia Australia; C/o Flinders Human Behaviour and Health Research Unit, Flinders University, Adelaide, Australia; The Bioethics Centre, University of Otago, Dunedin, New Zealand

**Keywords:** Community mental health, Community treatment orders, Coercion, Metaphors, Control, Patients’ perspectives

## Abstract

**Background:**

Community Treatment Orders (CTOs) are often complex because of the ethical tensions created by an intervention that aims at promoting the patient’s good through an inherently coercive process. There is limited research that examines the complexity of CTOs and how patients on CTOs and workers administering CTOs make sense of their experiences.

**Methods:**

The study involved in-depth interviews with 8 patients on CTOs and 10 community mental health workers in South Australia, to explore how they constructed their experiences of CTOs. Critical discourse analysis (CDA) was used to analyse the data, supported by NVIVO software.

**Results:**

Analysis of the interviews revealed that patients and workers experienced the CTO process as multi-dimensional, including some positive as well as more negative constructions. The positive metaphor of CTOs as a safety net is described, followed by a more detailed description of the metaphors of power and control as the dominant themes, with five sub-themes of the CTO as control, wake-up, punishment, surveillance, and tranquiliser.

**Discussion:**

Metaphors are a way that mental health patients and mental health workers articulate the nature of CTOs. The language used to construct these metaphors was quite different, with patients overwhelmingly experiencing and perceiving CTOs as coercive (that is, punishing, controlling and scrutinizing), whereas workers tended to perceive them as necessary, beneficial and supportive, despite their coerciveness.

**Conclusions:**

By acknowledging the role of metaphors in these patients’ lives, workers could enhance opportunities to engage these patients in more meaningful dialogue about their personal experiences as an alternative to practice predominantly focused on risk. Such a dialogue could enhance workers’ reflection on their work and promote recovery-based practice. More understanding of how to promote autonomy, capacity and supported decision-making, and how to address the impacts of coercion within care, is needed.

## Background

The concept of recovery is embedded within Mental Health Acts and recommended as central to the delivery of mental health services in many countries [1–3]. Recovery involves acknowledging the person’s capacity for agency; how they are enabled to maximize a positive sense of self as a citizen, and minimize threats to agency by what Fisher [4] described as ‘being done to’ within systems of care (p. 12). The conditions that make recovery possible, or can indeed thwart it, are constructed in the interactions between patients with mental health diagnoses and mental health workers (and the services they represent). Recovery is a concept that imbues philosophical or existential ideas associated with meaning making that may be less than straightforward to translate into practice in the treatment and care for people with mental health needs. There has been little research, however, examining how patients and mental health workers construct their roles respectively as recipients and providers of treatment and care, or the meanings they give to their actions within these constructions. In particular, there has been little consideration of how patients and mental health workers might use metaphors to make sense of their experiences. This is interesting, given that the concept of recovery is arguably a deeply personal notion involving ideas and experiences that might be difficult to put into words.

A recovery-oriented approach seems particularly important for those who are subject to assertive treatment via a community treatment order (CTO) (known as involuntary outpatient treatment (IOP) in the UK) that requires them to receive treatment in their home for their mental illness [5]. A number of concerns have been raised about the use of CTOs. These relate to their inherently coercive nature and the subsequent negative impact on civil liberties [6–8]. CTO have also been labelled instruments of social control [9], which seems ‘incongruous’ with recovery [10], and that may fuel a focus on risk that has then, “diminished the significance and legitimacy of therapeutic responses” [11] (p.287). CTOs place mental health workers in a complex and contradictory paradox in which caring whilst simultaneously policing are combined. Dunn et al. [12] examined these processes in detail, in particular, the process of making threats and offers to patients as a strategy to increase their adherence to treatment. CTOs might deter people from seeking help and engaging, hinder medication adherence [13], and reinforce negative stereotypes about people with mental illness as dangerous [14–16]. Other concerns are that the assessment of risk of future harm to self or others on which CTOs are based is unreliable and based upon weak evidence that they reduce risk [17–19].

Internationally, rates of CTO use vary between countries [20, 21]. This variability suggests that CTOs have become a default option in defensively oriented mental health services [22] (p.355). Fundamental to most Mental Health Acts is the understanding that people have the right to be treated in the least restrictive environment. However, research has shown that many people placed on a CTO continue to experience negative feelings about their involuntary treatment, and that it exacerbates their feelings of stigma and disempowerment [23–28]. Added to these concerns is a lack of evidence for CTOs producing positive clinical outcomes such as reducing rates of readmission of psychotic patients, increasing time to readmission, reducing the number or duration of admissions, or improving treatment adherence [8, 21, 29–31]. A recent study of 90 mental health patients by Suetani, Foo and Wilson [32] found a trend for greater compliance to depot medication by those not on a CTO compared with those who were on a CTO. This suggests that, while forced cooperation might lead some patients to accept medication in the long-term, other patients might construct and interpret the experience as a loss of autonomy and develop negative attitudes to medication and help-seeking from mental health services, more generally.

Newton-Howes and Banks’ [33] found that patients’ experiences of CTOs vary because, while many patients described greater coercion when subject to a CTO, there were also many patients who felt they were better off when their mental health was managed with a CTO. They called for further research to more clearly understand why these differences exist. The ways in which patients and mental health care workers navigate, negotiate and experience CTOs depends upon a number of factors, many of them interpersonal. Understanding how the meaning of CTOs is constructed (understood) requires paying attention to the first person accounts of those who have lived experience of this process [7, 32]. Current Australian mental health legislation appears to focus on the process of imposing CTOs, with little accountability for what workers and patients do during the CTO period. Understanding how each party constructs the CTO process might offer a better understanding of how to manage the tensions described above.

## Methods

The data presented in this paper was collected during a qualitative research project that explored the experiences of the CTO process for community-dwelling patients and community mental health workers in one state of Australia. A subset of the data is presented in this paper to examine how patients and workers understand and construct their experience of the CTO process.

The importance of consumer involvement in research is clearly established; it enhances its quality and appropriateness at all stages of the research process, from the development of initial research questions to dissemination of findings [34]. The conduct of this research was strengthened by the direct involvement of mental health consumer researchers. Both SL and AS are consumer and carer advocates within the mental health field with direct experience of mental illness and receipt of treatment and care from the consumer perspective. SL also has over a decade of experience as a mental health professional, working with people on CTOs, and AS has been subject to CTOs many times in the past; therefore, their input to this research was invaluable. Both SL and AS provided essential consumer input to the development of the research and provided important critical comment to the interpretation and analysis of data.

### Participant sample and recruitment processes

The participant sample included 8 patients and 10 workers. The patients were women and men aged 18 years and over living in South Australia. All were patients of State-funded clinical community mental health services, who were currently on a CTO and who had been so for at least six months. This timeframe was chosen to appease the ethics committee because it expressed concerns about interviewing patients on CTOs during the earlier stage of the order. This timeframe also provided a solid period of time (6 months) from which participants could recount their experience of receiving contact from mental health services whilst on an order. The patients were recruited via their mental health community case managers who confirmed each patient participant’s capacity to provide informed consent and to ensure a safe interview for participants and interviewers was confirmed. Exclusion Criteria for the patients were:

- an intellectual or cognitive disability that rendered the person unable to provide informed consent;
- current suicidality or other risk as determined by the mental health services; and
- a case-note alert signifying that two-person contact was required.

After receiving the initial invitation from case managers, most potential patient participants contacted the lead researcher directly. Some patient participants asked the case manager to provide their contact details to the lead researcher, and in these circumstances the researcher then contacted the patient and explained the research, providing the opportunity for the patient to opt in or out without the knowledge of the case manager.

The 10 worker participants were community treating doctors and community case managers who worked in the professions of psychiatry, nursing, occupational therapy and social work. All worker participants had been employed for 5 years or more, and had direct experience of working with people on CTOs. The workers were recruited via a general global email sent to the service’s clinical lead for distribution to staff.

### Data collection

All participants were interviewed individually using an in-depth semi-structured interview approach. To facilitate these interviews, an interview guide was developed in consultation with a project reference group, informed by the reviewed literature (see Table 1). The lead researcher (SL) conducted all interviews to ensure consistency and engagement with participants. Prior to the commencement of each interview with patients and workers, the research was explained, voluntary informed consent was confirmed and a consent form was signed. Patient interviews were undertaken either in their homes (*n* = 4), a public location where the patient felt comfortable (*n* = 2), or the lead researcher’s office (*n* = 2). All worker interviews (*n* = 10) were undertaken in a private office at the community mental health service, during their usual working hours, and at a time convenient for them. Interviews were audio-recorded and professionally transcribed verbatim, except for interviews with two patients who requested note taking only. Due to the potential for highly sensitive topics to be discussed regarding experiences of being on or administering a CTO, participants were offered support to link with existing structures or services (e.g., case managers for patients, or employee assistance programs for workers); however, no participant reported needing this assistance. Reflective notes were made after each interview to record the interviewer’s observations. All participants were provided with the opportunity to view and edit the transcript of their interview. The researchers met regularly to discuss the data and its potential meaning as interviews proceeded. Where possible, these sessions were audio-recorded to capture the dialogue.

**Table 1:** Worker and Patient Interview Guide

Workers
1) Describe what you think of CTOs for people with mental illness? Benefits? Concerns?
2) Describe your own experience of delivering treatment and care to clients on a CTO?
3) What factors do you consider in determining the level of involvement of the person and their decision-making capacity when applying for a CTO and/or providing treatment and care during the time that they are on a CTO?
4) Describe your experience of the Guardianship Board hearing process and of applying for a CTO, or providing input to an application to the Board?
5) What types of support do you provide to clients while they are on a CTO?
6) Are there circumstances that prevent you from providing the support you would like to provide to clients on a CTO? Explain?
7) What do you perceive as the impacts for clients of being on a CTO? Benefits? Problems? Impacts for you/the service/ others?
8) Are you involved in the development of mental health care plans for clients on a CTO? If so, your experience of these and processes followed? Client copy? How often reviewed? Your perception of what clients think about them?
9) How could MH services improve how they provide support to people on a CTO?
10) Do you have any other comments to make about your experience of providing treatment and care to people on a CTO?
Patients
1) Description of how you came to be on a CTO? How long? Others? Recollections of interactions with mental health staff and Guardianship Board hearing?
2) Description of your experience of receiving contact with MHS since being on a CTO? Case manager? Psychiatrist? Other support people?
3) Level of involvement in making or sharing decisions about your treatment since being on a CTO? Examples? How you felt about this?
4) Level of involvement in making or sharing decision about other parts of your life since being on a CTO? (eg. Psychosocial support needs). Examples? How you felt about this?
5) What support does the mental health case manager provide to you as part of their contact with you?
6) What do you perceive as the impacts for you of being on a CTO? Benefits? Problems?
7) Do you have a mental health care plan? Your view of it? Have you seen it/got a copy? How often is it reviewed with you? Your involvement in its review?
8) Do you feel that your life has changed since being on a CTO? Why? Why not? If so, what has changed?
9) How could mental health services improve how they provide support to people on a CTO?
10) Do you have any other comments to make about your experience of being on a CTO?

### Data analysis

The influence of the qualitative approach is demonstrated by the development of detailed, ‘thick’ [35] and integrative analyses as part of the research [36, 37]. This kind of analysis is vital in order to understand the complexity of the CTO process, and the experiences of those who participate within it.

Initially, four randomly chosen interview transcripts (2 patient and 2 worker transcripts) were open coded by the researchers independently. The researchers then met to discuss and debate their assigned codes to establish an agreed coding framework. This framework was used to code all remaining interviews with the assistance of NVivo 10 software. Following an initial round of open-coding, selective-coding was applied to identify key themes in participants’ discussions, as well as commonalities and differences across the transcripts. Once approximately three quarters of all interviews were coded, the researchers met again to discuss and determine core and sub-themes. During this process it was identified that several participants were explaining their experience of the CTO process in explicit detail and in ways that indicated that the participants were making sense of what had happened to them by using metaphors and other narrative techniques to construct an account of what happened during their psychiatric care. Multiple participant constructions of the process were evident and the researchers began to discuss these and the implications that participant constructions might have as part of the CTO process. In particular, the presence of metaphors to describe their experiences was particularly striking in the consumer interview data; prompting us to examine these constructs in more detail.

Mould, Oades and Crowe [38] state that metaphors can, “provide a bridge between subjective experience and clinical descriptions” (p.283). They, “convey information about the constructs people use to perceive, experience and portray their world” (p.286). They allow us to form and express concepts for defining our reality for ourselves, and describe our experiences to others [38]. Metaphors are often used when we are trying to find a way to express something that is difficult to put into words. Using the basic definition, metaphors are figures of speech, present in everyday language; they often go unnoticed and serve to represent or symbolise something else that the person is attempting to convey [39]. Carpenter states that the use of metaphors in qualitative research can help to understand familiar processes in a new way; to illuminate meaning of experience by being, “a powerful strategy to portray complex realities…and adding depth of meaning and understanding” (p.275) [40]. However, they caution that using metaphors, “should not become a self-serving attempt at creativity that supersedes subject and substance” (p.274) [40].

To understand the reasons for these multiple constructions, and to develop a rigorous analysis of this aspect of the data, the critical discourse analysis (CDA) method was employed. CDA is informed by broad understandings of discourse, extending beyond linguistic analysis of words. CDA emerged from social theories, particularly theories of power, which view discourses as the active and constructive components of all social interactions [41]. Discourses operate within social actions to produce meaning, shaping the words we use, ideas we convey, practices that we select and explanations that we provided [41]. Our specific method of CDA was informed by the work of Hart [42] who considers the power of metaphor as an analytical tool. Hart provides a conceptual framework that can be applied to analyse text to reveal how particular understandings of a process might be conveyed by participants through the use of metaphorical constructions. Hart’s framework prompts researchers to question the references that participants make, and to critically examine the *ways* they describe their experiences, to understand the systems of meaning that are operating behind their explanations. Applying Hart’s framework helped us to understand the meaning being conveyed by participants when they used metaphorical language such as “The CTO process feels like…” or “Administering a CTO reminds me of …” or “Applying a CTO makes me worried about…” Statements such as these helped us to understand the conceptual framing evident in the participants’ narratives and to see how these framings could provide insight into the meanings that the CTO process holds for them.

## Result

Analysis of the interviews revealed that patients and workers experienced the CTO process as multi-dimensional, including some positive as well as more negative constructions. The positive metaphor of CTOs as a safety net is described, followed by a more detailed description of the metaphors of power and control as the dominant theme, with five sub-themes of the CTO as control, wake-up, punishment, surveillance, and tranquiliser. Pseudonyms are used throughout.

### CTOs as a safety net

An example of a more positive construction is the way that five participants (two patients and three workers) described CTOs as being akin to a safety net that assists in preventing the possibility of worse outcomes for patients. One patient explained that he felt reassured that workers would be available to provide the support that he required if he was to experience an episode of mental illness that caused him to lose perspective:

*(John-patient) You know knowing that if you do get sick and that you guys can realise ‘Hey you've gone off your meds and that and you're getting sick’…‘Hey we can grab you and bring you in and you know get you back and on the right path again’.*

One worker employed the argument of a safety net as a way of dismissing patients’ more negative constructions of the CTO process:

*(Kim-worker) I've heard people talk about, “But I haven't done anything wrong”, seeing it as a bad thing or a punishment and I like to think of it more as more of a safety net to ensure they have some support or contact.*

It is clear that the positive framing employed by this worker helped her to still apply CTOs, even when patients projected or expressed negative feelings about the CTO, because she could look past these to see the benefits that she believes to be associated with the process. This, as well as other references made during the interview, helped her to reconstruct the CTO as a positive tool for patients, to justify the use of coercion, rather than as a more negative imposition of power.

### Metaphors of power and control

There were also interconnected metaphors that were primarily negative and expressed a controlling and disempowering experience of being placed under a CTO. A number of these linked metaphors are present in the observations of one patient.

*(Jenny-patient) Yeah, like there’s not enough collaboration with the person, like compromise…even when I’ve been in extreme distress and I’ve been, you know, like whatever they describe as psychotic or being suicidal or whatever, I’ve still got my reason. I’ve never completely lost my rationality or my reason…but then they just come up and drag me and put me on the ground and force me to the ground, and there were bruises all up and down my legs. You know, like, they were just too heavy-handed…the police don’t know how to deal with mental health problems. They just-brute force-it’s just brute force…I call it being incarcerated or jailed because that’s what it is…I’ve been chained and shackled to a stretcher…sometimes you get someone that they’re nice to you, and then they change and they suddenly start being nasty to you, and it’s like, ‘What did I do wrong?’ you know…when I was locked in the lockup, I was detained and I was terrified of this man next door because…he just seemed like scary to me and I was worried about being raped to be honest in the psych ward because I think if it happens, who’s going to believe me, you know. Like, she [the nurse] was threatening-they were threatening to call the code black…because I wouldn’t take a drug, because I wanted to be awake at night in case he tried to come into my room…I was very upset and anxious because-and I couldn’t sleep. For a few nights there I just sat against the bed and I would just be bolt upright in case-waiting because if he come into my room. There’s a lock on the door but they often-when they come in to do your checks at night, they leave it unlocked. Sometimes they leave it unlocked, and then anyone can get in… How could anyone be happy on one of these drugs, you know?…Being on a CTO? It’s like being in prison in your mind.*

In the above quote, Jenny articulates an experience where she felt vulnerable, threatened but also one where her agency, while unwell, is controlled and repressed. ‘Loss of control’ or ‘disempowerment’ was expressed by a number of the patients and they used metaphors to express the nature of these. Jenny describes her experiences in a way that suggests they felt punitive, or like a form of punishment for a failure to be compliant and that is similar to what other patients said. They used a number of metaphors of control and we have organized the findings under the following sub-themes:

- CTO as control
- CTO as a ‘wake up’
- CTO as a punishment
- CTO as a tranquiliser
- CTO as surveillance

### CTO as control

Underlying many comments by patients was the view that CTOs were ultimately used by workers to control them; as a means for workers to assert their authority. The belief in mental health services having a helping, therapeutic role was absent from these patients’ descriptions of their experience.

*(Joan-patient) It’s a power trip. That’s all I can put it down to. He likes to be in control of my life and he’s not…he is supposed to be the therapist and the controller of the CTO…I don’t know why [doctor] is persevering and determined to put me on a CTO. He doesn’t know me. I haven’t had proper consults with him for a long time, and the consults that I did have with him he says, ‘I’ll do what I want to do’…I’m being controlled.*

*(Thomas-patient) And I think that’s what was hard for me to get over, that idea that I was having someone tell me that I had to do something, you know, that element of control over my choices…I just felt like I was being put in a prison that had like…invisible chains, like you couldn’t actually see them, whereas you go to gaol they put chains on you…I just felt like there was this invisible sense of, you know, like doom coming over my life.*

They described the core of this problem as resulting from workers’ refusal to listen to them and understand their perspective.

*(Peter-patient) I had problems that weren’t medication problems but he would not let me explain.*

*(Jenny-patient) Might be it’s true that they prefer to do it voluntarily, but they do it by force and it’s like there’s no dialogue. When someone’s forcing something on you, there’s no dialogue.*

Workers were aware of their role in imposing treatment on patients, though it was less clear that they understood fully the impact of this on patients. They provided a range of justifications for why their role meant they should take control. Some, like Judith, expressed complete comfort, explaining that his/her actions were for the future benefit of patients, because patients’ did not have the capacity ‘to make any reasoned decision on their own behalf’ whilst unwell, that all of their decision-making was ‘forfeited’. Others, like Roxanne, seemed to struggle with the justification of control, offering a range of reasons that seemed confusing and contradictory at times. Some, like Kim, seemed more distant and dismissive of the impacts on patients.

*(Judith-worker) Well I can recollect hearing about the introduction of the first of the community treatment options just after the-when the major tranquilisers started to become on the market…it was quite world-breaking legislation…the fantastic ability to be able to insist on treatment without taking people to hospital, leaving them in the community and be able to enforce a degree of adherence. I just think it’s fantastic…they might even have some insight but the unwillingness to take medication-and then you have other people without insight, unwilling to take it and the consequences for them are really very, very unpleasant…I feel that one only does this as a last resort but I also believe strongly that people do have the right to be treated and that if we expect someone without insight to behave rationally then I think we’re expecting something that is just not going to happen…someone sort of quite paranoid they cannot see that they really do need these meds then of course really are in no position to make any reasoned decision on their own behalf, in my opinion.*

*(Roxanne-worker) So I would rather carry that path [seeking cooperation from the patient] first but, however, there are times when you just really need to take that control away because they’re not well…being on somebody else’s journey; it’s not our journey to determine, it’s theirs, and it being recovery-based…I suppose they feel powerless really a lot of the time, so it’s understandable that they want to be in control, yeah…I’d hate to be on a Community Treatment Order.*

*(Kim-worker) Unfortunately I think a lot of the time also consumers see it as the medication, which is a big component of it, and think that they have to have exactly what their doctor prescribes…they can obviously negotiate things, but I guess a patient does feel disempowered often.*

Some workers acknowledged that their peers might hold a range of views about their role in enacting a CTO, with some being more likely than others to resort to exerting control over patients. Geoff’s comments suggest that overt coercion was a usual part of how some workers delivered care.

*(Geoff-worker) To me, it’s about what is the impact of this person not receiving treatment on their life, on their career opportunities, on their opportunities for relationships. I suppose my general principle is I think there are some people who use the CTO as a big stick. ‘If you don’t take your medicine, if you don’t have the depot, we’re going to call the police and get them to bring you into hospital’.*

Others, like Tim, made comments that suggested that there were inherent flaws in the CTO process that fostered more controlling responses by workers and expectations by patients of that control as the dominant response by workers. His comments suggest that he saw a greater focus on developing workers’ therapeutic skills in engaging patients as one solution to this dilemma.

*(Tim-worker) CTOs are really only good for one thing which is administering medication by injection…you’re empowered to inject somebody with medication if they’re in your presence. It doesn’t stop somebody from hiding or running away…if their psychosis were to be uncontrolled, be at risk of acting very dangerously and neglecting their health or failing to eat or drink, but if they accept the medication makes a difference for them with whatever rationale underpins that, then they still might not need a CTO…You certainly don’t need all three for somebody to accept medication. It might be that the medication helps them sleep better, it might be that they feel less anxious, it might be that they’re less sensitive to the voices which are definitely being broadcast from a mechanical device when they have the medication….We’ve trained people that, you will only stay in hospital if you’re on an ITO [Involuntary Treatment Order], you’ll only have your medication if you’re on a CTO, rather than finding any other point of common ground to negotiate these things.*

### CTO as a ‘wake up’

Three patients used the terminology of a ‘wake up’ to describe the impact of the CTO process within their lives. The patients used this term to explain how the CTO helped them to develop a new perspective on their lives, to stop and consider, and to assist them in developing behaviours that might contribute positively to their mental wellbeing.

*(John-patient) You come in the hospital and it's a wake up call…it's like you know I've been on medication for a number of years. Then you think to yourself, you know, you get admitted to hospital then it gives you a bit of a reality check. You say to yourself and that ‘Don't do this again because it's not worth it’.*

Use of the term ‘wake up’ expresses the idea that a CTO is a powerful, perhaps sharp, mechanism that can ‘jolt’ participants from thinking and behaving in particular ways. In much the same way that a loud alarm next to the bed can jarringly pull us from a restful sleep, a CTO might jolt a patient into seeing that something must change. This construction was softened by workers who instead spoke of the process as if it motivated patients to seek helpful contact with workers. Although the three patients also used the term ‘wake up’ to explain how the CTO process helped them, the softer tone of motivation reflects a key difference in how the worker and patient participants understood the process; with patients focussing on the sharpness of the approach while workers focussed more on the ability of a CTO to facilitate change, as demonstrated by the following two workers’ comments:

*(Kim-worker) Some people tell us, as well, they'll only take medication if they're on Community Treatment Order; and there are a number of patients that seem to have ongoing ones, and they've said, “Look, if it stops, I'm just not going to take it” and they’ll be frank about it but in their head as well if they're on the order they'll come in without-not even thought about having the police coming to pick them up…they feel mandated to some way, which is quite disempowering but I'm not sure how they perceive that.*

*(Jane-worker) One lady that I’m thinking of says, “Well I don’t think it really makes any difference but I can’t be bothered arguing about it too much so I’ll come in if there’s something that says I have to, but otherwise I won’t because it doesn’t make much difference to me”.*

These two quotes also reveal that the workers spoke of the CTO being a necessary (and often welcome) facilitator because patients were otherwise stubborn or uncooperative. The patients’ need for a ‘wake up’ conveys a different understanding, however; one that contradicts the assumption of a deliberate lack of cooperation and implies the patients’ perceived need to be supported in taking a different path or in understanding how a change in behaviour could be possible or beneficial. A ‘wake up’ is also fundamentally about control and power, it’s a technique that can be a catalyst for positive change but it is a ‘metaphor of control’.

### CTO as a punishment

One of the most dominant constructions of the CTO process related to the notion of punishment. Over half of the patients expressed the perception that CTOs can be imposed as a form of punishment, or admitted that they believed this at some point in their experience. Some patients spoke directly of circumstances where they felt as though a CTO had been imposed to punish them for behaviour that contradicted the instructions of workers by using words such as Joan who said, “…you don’t get no second chances.”

Other patients recounted experiences where they had been threatened with a CTO as a means of encouraging them to adopt particular behaviours.

*(Jessica-patient) The key worker said if I wanted another support worker, then I needed to stay with mental health services. Like it was a threat they were using against me.*

Jessica’s observation looks like one we should take at face value: that appears to be a literal account of an experience that felt like punishment. Workers also recognised the potentially punishing and threatening implications of the CTO process, and of their own behaviours as part of implementing the CTO. Two workers mentioned that they actively tried to reassure patients that they were not being punished, and in doing so conveyed some discomfort with their role in imposing care.

*(Kim-worker) Like sometimes, you do think it's kind of like a punishment. So like, "You haven't done anything wrong. You haven't. This is the reason", and I'll explain.*

Another worker expressed frustration at the mental health system which forced him to work with, and try and navigate through, colleagues who might use the CTO process to threaten and punish. This worker’s discomfort appeared to create the potential for conflict in their professional relationships and it influenced their approach to practice:

*(Geoff-worker) I think there are some people who use the CTO as a big stick; ‘If you don’t take your medicine, if you don’t have the depot we’re going to call the police and get them to bring you into hospital’. I don’t think I’ve ever said that to anybody…one of our registrars the other day …said he was going to go back and say to the patient, ‘You can go home and you can pick up some clothes but if you don’t come back we’re going to send the police and they’ll bring you back to hospital’. I said, ‘Well, that’s threatening, and I don’t think we should be threatening people’. My general approach…is to try and say, ‘This is a safety net’ and to try and articulate the reasons why I think there needs to be that safety net, but also understanding that people might not agree with that and that’s fine, but in terms of my general practice…it’s never just about the individual, it’s about the context that they’re embedded in, so you’re supporting all the people around them and explaining to them as well what’s happening.*

Using the CTO as a ‘big stick’ is a fairly clear case of a coercive threat, i.e., unless you do this we’ll need to do something that means you have to. This is also an instance of ‘CTO is punishment’, which seems quite literal: there is no need for a metaphor to convey the meaning of that experience. Another element of the punishment theme is the way in which a consequence can quickly follow a transgression, in the same manner that it can when trying to teach a child.

*(Vicky-patient) See I should have gone back to my original Psychiatrist and he should have said, ‘Well all right. You lied. So what!’ I mean, it doesn’t need to be done by Order; you’re voluntarily willing to go back…that opportunity wasn’t given to begin with so an Order was just put in place…I might be volunteered to it [injections] or…gone back onto tablets but there was no trust you see. There was one mistake and they…that’s like disciplining a child isn’t it, and I’m not a child, I’m an adult, and we do lie [laughing].*

Controlling or disempowerment was also reflected in themes that are more clearly metaphorical than punishment. Some patients experienced the CTO as something that negated their agency, rather than a process that might lead to underlying issues or problems being resolved.

### CTO as a tranquiliser

Another dominant theme, evident in the comments of most patient participants was that the CTO process offered little therapeutic value to them because it was perceived to be used largely as a means to tranquilise patients so ensure their cooperation, leaving their ‘real’ concerns unaddressed.

*(Jenny-patient) Basically, they’ll see me the way I am now…I’m calm so that’s good apparently. They don’t see what’s on the inside which is that I’m really depressed, apathetic, emotionless, I don’t enjoy my normal interests. So basically, they’re just going to see a drugged up zombie basically, and think…‘Oh right, well this person’s calm and this is controlled’.*

This use of CTOs as a tranquiliser was perceived by patients to have negative consequences; in particular, it denied their agency to participate in their own care, to feel listened to by workers, even when they wanted to engage with workers to overcome their problems. As well as calming somewhere, the medication and process of a CTO could tranquilise their agency.

*(Joan-patient) Knowing that I had the depression and the fears and the traumas I’d been through, and I couldn’t hold it anymore…I needed someone to listen to me. Even police would not help me…But the thing was I wanted to talk…Even the doctors at [hospital] wouldn’t talk with me [When asked why she thought that was] I don’t know, I’ve got no idea. I would talk to the student psychiatrist and tell him everything…But there was nothing…It’s like [doctor], just-I’m full of all this stuff, I want to tell somebody and all they do is inject me, medicate me and hold me in hospital for six weeks. It doesn’t make sense; it doesn’t make sense. Sorry.*

Another patient participant, Vicky who was in her 50s and on her first CTO, talked openly about her desire to engage in better physical health, She appeared to have thought a lot about how drinking and smoking was affecting her and expressed the desire to take control of these ‘drugs’. She had made a decision to deal with these by also stopping her medications, which she perceived also as ‘drugs’. She described her experience of being placed on a CTO, at length, stating that she was now quite willing to take tablets, and that she had a good relationship with her existing doctor. But, the inpatient doctor had put her on a CTO anyway despite her being open to them that she had stopped her medications and that she had lied about this initially. It seemed that the potential to engage this person was there ready for workers to take advantage of, but they did not do that.

Another patient’s description of her experience also suggested that there was opportunity for workers to engage in collaborative dialogue with her. Instead, she felt that they were not interested in this option; choosing to use medications as the first option in their contact with her, and to address her expressed needs through the use of tranquilisers, tranquilising her as an agent, instead of other recovery oriented approaches.

*(Jenny-patient) There’s no-it’s just, ‘you’ve got this disorder’, bang, ‘you’re on this drug’, bang…the only one that can take me off this drug or ameliorate it…is a psychiatrist. The psychiatrist who gave me a prescription for - I have this side-effect thing that I’m on because of the drug, and I have to take Benztropine or Valium to cope with it…They were giving me scripts for yet more drugs to cope with the initial drug, and it just keeps on going on, like, you have to take a drug for the drug for the drug….There’s no input given…they don’t want to have a dialogue with you, they don’t want to listen to you, how badly it’s affecting you, how much pain you’re suffering. You know, they don’t want to listen to that. All they want to do is just drug you and then send you on your way…I have kind of said I don’t really want anything to do with her [the case manager] because…I don’t want to be on the drug at all. My view is what’s the point trying to work with them when they’re just trying to drug me?…You can be as intelligent and articulate as you like, you can even be as calm and rational as you like and you’re still going to get drugged, still going to get put on a CTO because that’s the way they work.*

The converse perspective was presented by several workers who described medication as the first line of treatment while they waited for the patient to gain some insight, and waited for engagement to develop once the drugs started working. Workers talked about CTOs being important to move through a crisis by ‘taking the edge off’ of the situation.

*(Kim-worker) If you’re forced to use a CTO in this situation, you often are buying time to try and stabilise all the social parameters in that person’s life, and it will give you that chance to stop that particular bit of the poor situation and so you can start to actually work with people…one thing I’m very much aware of is, that people-at least in the earlier stages of serious mental illness-frequently do not have the luxury of having insight and being able to understand what is happening to them.*

Jenny’s experience is deeper than merely being administered sedating pharmaceuticals; what she describes can be read as a tranquilisation of her as an agent. Her perception was that wish to be in dialogue with the service providers was ignored and she was tranquilised, as if they didn’t want to hear what she had to say. She made other comments that are consistent with this construction. She describe her CTO experience as one

*(Jenny-patient)…where you’ve got no rights, no autonomy…there’s no way you can defend yourself, you’re just completely a victim…there have actually been reported events of women being raped in psych wards. It’s happened in Victoria…I just don’t feel safe there. If I was being forced to see him [doctor], that’s when it’s like ‘You’re not going to get me to talk because, like, I’m talking to you because this is voluntary; this is completely voluntary on my part’. I initiated it, but on his part it’s like-when I’m forced to go there and I have the police threatening to come into my house and take me away if I don’t - it’s like, when you treat me like that, like an animal, like a criminal, I’m like, no way, I don’t want any dialogue with them.*

For Jenny, the CTO not only resulted in her being sedated and medicated but her ability to be heard, to be safe and her rights, were ‘tranquilised’ by the process. This metaphor associated with power and control is similar in force to what a number of patients said about the ‘surveillance’ nature of CTOs.

### CTO as surveillance

Several patients described the CTO process as like being ‘locked up’, being ‘treated like a criminal’, even though they were living in the community, with adverse effects for their perceived freedom and autonomy, generally.

*(Joan-patient) I don’t want to be reminded about being locked up. I wasn’t ill enough to be locked up. I was depressed and stressed, and nobody-they just locked me up, there was no counselling in the hospital, only medication…I feel like [doctor’s] treating me like a criminal because he got police to break into my premises…He wrote false statements to the Guardianship Board about me. He really is treating me like a criminal…I really believe CTOs are for people that have done something wrong and need to be looked after, yeah. I do feel like a criminal…especially after being in prison, because I’d lost-in 6 months I lost 30 kilograms because I wouldn’t eat. They had me-I preferred to be locked up in a D division cell [maximum security] than be abused and all the shocking stuff that prisoners do to other people, but that-yeah, that was pretty horrific.*

One patient described her experiences at length and the impact of the CTO process on creating an overwhelming sense of disempowerment. She seemed able to articulate some very fundamental issues about autonomy and respect. She described quite reasonable fears that were met with coercion by workers as the first option.

*(Jenny-patient) I associate her [the case manager] with being violated in my own home. Like, I’m happy to have you in my home because you asked permission, you didn’t force your way in here. You respect my autonomy and my right to decide for myself, you know, who I want in my home…Yeah, and then when you have people, like, police and stuff violating that stuff by breaking your-they don’t break your door but they’ve got a key apparently that they can just use to get in any time. It makes me feel scared and insecure and I just-I hate them, you know, because they just-they’ve broken into my home and they drag me out of here against my will and, like, I don’t believe that’s acceptable.*

Workers described the CTO process as imposing increased surveillance as the dominant component of ‘care’, even when this had not necessarily been their intention. They gave a range of reasons for this. Some, like Laura, actively tried to resist seeming dominant in their interactions with patients. Others, like Tim, saw it as an inherent part of trying to help some patients with severe psychotic disorders, a right to treatment that patients ‘deserved’ and that workers had a ‘duty’ to provide, and sometimes a necessary component to the recovery process.

*(Laura-worker) If you get pieces of paper out, people don’t like it…So every time I meet people, every 3 months, it’s mandated as a KPI [key performance indicator], but just generally every time I see people we make some kind of link to the care plan because it’s the interventions that we’re doing, not just from a CTO perspective but what does the person want from me and where do they want to be in, you know, a year’s time.*

*(Tim-worker) Mental health clinicians get habituated to depriving people of their liberty because we are so convinced that we are doing the right thing, and we have a sense of having a finite number of tools with which to do the right thing by people, or you just don’t think about what it means to have your choices taken away, and I think people chaff under that…really it means having medication, turning up for the occasional injection and 160 hours of your week is still your own, but that sense of being confined does matter…there’s the really unpleasant end of the enforcement of CTOs around emergency services, police and ambulance and mental health staff turning up on your doorstep, everyone in the neighbourhood knowing your business, people seeing you are being physically restrained and cuffed in front of your house which is absolutely awful, and the burden of medication itself, so those are all the negatives of a CTO.*

*(Tim-worker) If effective treatment really can’t be delivered in non-coercive ways then there are people who absolutely deserve a trial of treatment, people whose lives will otherwise be blighted by psychotic disorder, in particular, who will be permanently disabled and it’s a vicious cycle…not to discount the principles of recovery at all, not so that people with chronic, severe psychotic disorder can’t find quality of life but, that the people we miss at the early points who don’t get it treated and they could return to a relatively higher level of functioning, the extent of disability caused by that I think, if that’s offset by a period of involuntary treatment then I will lose no sleep over that equation.*

Patients also perceived the CTO process as imposing increasing surveillance by workers on many other aspects of their lives. The negative impact was described as a global one in which all of their movements and decisions about other aspects of how they lived their lives were now open to question.

*(Jessica-patient) The CTO is about enforced contact with Mental Health Services, every other day. I have to answer the door, answer the phone and see their psychiatrist whenever they say. I was being discharged from hospital one time and I wasn’t even able to go home first because I had a doctor’s appointment that they made me go to.*

*(Jenny-patient) Someone rang the police and then the police rang [mental health emergency team] and then they just come around here. The reason why I had the police come around the most recent time was because I hadn’t been to an appointment with the psychiatrist…they threatened to take me to hospital if I didn’t show them my drugs. So I was forced to show it to them.*

## Discussion

The results of this study reveal that patients and workers use a range of metaphors to describe how they construct and experience the CTO process. Their constructions, demonstrated by the language they used, were quite different, with patients overwhelmingly experiencing and perceiving CTOs as coercive (that is, punishing, controlling and scrutinizing), whereas workers tended to perceive them as necessary, beneficial and supportive, despite their coerciveness. Of note, patients appeared more likely than workers to use metaphors to help explain their experience and their feelings, and to express their suffering; whereas, workers were less likely to use metaphors when describing their experiences, or to recognise the purpose of their use by their patients. Instead, there appeared to be more literal. For example, the use of metaphor by John who described ‘being on the shelf’ was striking; though many other examples were equally striking. Workers spoke in more literal and clinical terms, based in the ‘here and now’ of symptom management and promotion of patient compliance, which arguably then limited their concept of recovery-based practice [10]. This difference might exist because, for patients, the experience represents a more direct and complex reality [40] than for workers; that is, patients experience the full impact of a CTO at a time when they might not have all of the resources or capacity needed to put that experience into more straightforward language. Mould, Oades and Crowe [38] provided a detailed discussion of the use of metaphors by people with psychotic disorders. They highlight a fundamental discrepancy in the metaphorical language that such patients use to describe their personal meaning and experiences when compared with workers’ tendency to prioritise symptoms and to perceive such language by patients as a lack of insight. Andreason [43] argued that this situation can lead workers to miss an opportunity to engage patients in a more meaningful dialogue about their personal experiences. Such a dialogue could enhance workers’ reflection on their work and engagement of patients, and would be fundamental to recovery-based practice, given that many core features of definitions of recovery in mental health include notions of finding hope, meaning, identity and purpose [44–47]. Empathy has been identified as a key skill for adopting a recovery-orientation to mental health care [48]. An understanding of how and why patients on CTOs use metaphors would be useful for empathy because it would help workers to understand how it is for another person. The implications for reflective practice are significant and Brophy and McDermott [49] emphasised the importance of workers reflecting on imbalance in power inherent in their practice. A number of workers in our study did not appear to enter that space at all, which has concerning implications for their capacity for being empathic and reflective practitioners [50]. It is also concerning, given it might reflect an inability by some workers to ‘go to where the patient is at’ in understanding how patients on a CTO are expressing their experiences of care through the use of metaphors. This would likely hamper these workers’ ability to engage such patients and develop their trust during the CTO process that requires such skills to be arguably at their sharpest in order to provide effective person-centred care to this group.

The contrasting and often conflicting views expressed by patients and workers in this study are not surprising given the CTO process and concerns about coercion that have been raised repeatedly in the literature on CTOs [10, 12, 51, 52]. According to Mfoafo-M’Carthy & Williams' [10], definitions of coercion, “have one thing in common. They emphasise that power can be used to eliminate the prerogative of people who do not have the power to resist” (p.71). The results, especially the different views expressed about control, demonstrate the importance of understanding how patients experience and understand the CTO process, often as something bigger and more significant in their lives than workers might realise. How patients and workers understood the CTO process then impacted on how coercion was experienced, how they participated in the process and what outcomes they expected from it.

Patients’ and workers’ perceptions fundamentally defined their relationships. Workers’ descriptions show that they were attempting to deliver treatment through the lens of compliance. However, the solution available to them (the CTO) was undoing the desired outcome of the person engaging and being compliant with treatment. For some patients, like Jenny and Jessica, this meant that they developed a deep-seated mistrust of service providers and a fear of mental health treatment settings, generally. To them, workers represented authority, control and surveillance; with the consequent threat of disempowerment evident in several patient participants’ comments. Others, like Peter and John, seemed to be already disempowered, to have succumbed to workers’ control. Patients clearly described a sense of having their concerns dismissed, of not being listened to by workers. This lack of engagement with patients meant that a number of workers were drawn into a coercive role as their first response to patients who resisted their views of what was needed. This drew them into practice which resorted to making threats and offers to patients on CTOs [12]. This study’s findings therefore are in contrast to those of Struen et al. [53] who concluded that more of their CTO participants stated that they did not feel coerced. Differences may relate to differences in mental health systems between Australia and Norway, or nuanced differences in the sample and how they were recruited.

Ayra argued that, “It is only when the patient is unable to make an autonomous choice that the health professional has a duty of beneficence”, to act in the patient’s best interests [9] (p.474). However, seeking a CTO based on best interests without considering the patient’s capacity is problematic, not least because what the patient perceives subjectively as in their best interests may differ from what others perceive to be so, especially when there is evidence that it is made from a reasonable perspective [9]. We argue that Ayra’s argument needs to be unpacked more; that workers always have a duty to act out of beneficence. The distinction here is between strong and weak paternalism; the latter is more readily justified because it involves overriding wishes when autonomy is lacking, but even when someone clearly does lack capacity we should still be talking to them about what they want. A number of patients in our study recounted circumstances that appeared to demonstrate their capacity for quite rational thinking. Jenny’s concern about being raped in the locked ward was one of these, as was Peter’s desire to talk to someone about his emotional state. However, workers’ responses left little room for them to account for these capacities in their interactions with patients on CTOs. We argue here that capacity is not that crucial; both Jenny and Peter should have been listened to, regardless of their capacity.

Workers’ responses are likely a feature of the safety/risk paradox inherent in mental health care, which is shaped strongly by Mental Health Acts that take little account of working with patients’ capacity to contribute to decisions even though they may lack insight and capacity in some aspects of decision-making. CTOs exist within a complex and contradictory paradox in which caring whilst simultaneously policing becomes complicated. However, our results demonstrate that, by acknowledging the role of metaphors in these patients’ lives, workers could demonstrate that they are listening to patients; and that despite patients’ diminished capacity at the time, they should be listened to. Workers might not be able to empathise with the person’s experience of psychosis; however, understanding patients’ use of metaphor offers an important therapeutic vehicle for helping patients who experience psychosis to rebuild their sense of self [38] and, therefore, engaging such patients as an alternative to practice predominantly focused on risk. As stated by co-author AS during the data analysis period, when you experience psychosis, not only do you lose self-agency, you lose who you are as a person. It is more than a lack of control, more than being tranquilized; it is a loss of you. Some worker participants (for example, Judith) justified taking control where they deemed patients lacked capacity to make any decisions about their lives; Others like Tim argued that early CTO use was justified to prevent long-term disability, future harm and institutionalisation [10]; that people have a right to treatment. However, these accounts seem to stretch the sense of rights because the essence of a right is that you can waive it, but these arguments seem to be based on a right to treatment you cannot waive [12]. They also deny patients’ capacity to make some decisions, even when extremely unwell. For example, a detained patient can still know that they like chocolate not vanilla flavoured ice-cream, or still hold a preference for which political party they want to vote for [54].

Sawyer [11] argued that the growing focus on risk has altered the role of mental health workers as, ‘psychiatric risk managers’ and that this has, “diminished the significance and legitimacy of therapeutic responses” (p.287). Dunn et al. [12] examined these processes of making threats and offers to patients as a strategy to increase their adherence to treatment. Fishwich et al. [55] examined the caring and custodial tensions inherent in workers’ practice. This is further hampered by dialogues about risk and dangerousness which further work against reform because they give power to those assessing risk without constraints, which then distort mental health workers’ and the community’s cultural values about people with mental health issues. Several participants in our study confirmed these concerns. The experiences of disempowerment recounted by patient in this study exemplify what Foucault [56] called ‘power/knowledge’ which combines into a unified whole, “the deployment of force and the establishment of truth”(p.184) about those undergoing examination, determining the state of their health, and therefore justifying control of their behaviour through enforced treatment. Foucault described inherent power systems through the creation of norms that in-turn form the basis for knowledge. Within this knowledge production, the patient becomes ‘the case’ and caring is inherently an opportunity for control. The metaphor of surveillance described by patient in our study parallels Bentham’s Panopticon described by Foucault as the ideal architectural model of modern disciplinary power [56].

The dignity of risk is a core aspiration of recovery-based practice [46, 47, 57]; however, the means to apply this within routine practice when working with patients on CTOs seemed unclear for many workers in our study. They were legally bound to enact CTOs, but are also required to establish a therapeutic, person-centred relationship to engage the person in recovery-oriented outcomes. Jenny exemplified patients’ desire to be treated with dignity and respect by workers. This desire is reported by others exploring the views of patients on CTOs and their desire to be heard, involved in decisions, and given information about their care; whereas [58–61]. Glenister [62] described this tension in the context of mental health nursing as existing, “in the moral grey zone between caring and controlling.” (p.50). Several workers talked about their duty to provide enforced treatment when patients did not have capacity; describing such patients as ‘deserving’, suggesting a benevolent paternalism shaped by their constructions. For some workers, like Judith, there was no ‘moral grey zone; patients on a CTO forfeited their capacity to make decisions completely. Patients’ capacity was therefore something that would come later, once the person developed insight, and separate from the workers’ role with patients. This left workers to rely on medication compliance, usually administered coercively to patients, as the frontline of care, especially in the early months of the CTO. Care based on these dominant constructions left little room for therapeutic engagement. It unwittingly drew workers on a path of what Szmukler and Appelbaum [51] described as persuasion, interpersonal leverage, inducements, threats, and compulsory treatment. This involved either ‘hard paternalism’ (actions taken in the patient’s best interests without their consent, where the patient believes they could make their own decision) or soft paternalism (actions taken only if the patient lacks decision-making capacity, where treatment is in their best interests). Either way, patients’ capacity was denied, and they perceived that workers were not listening to them, whether patients had capacity or not.

The ‘tranquiliser’ as the only early option for some patients, given their level of ill-health or risk, has adverse consequences for the therapeutic work that needs to follow to assist the person to manage their mental health in the longer term. Patient participants were clearly seeking dialogue with service providers in an attempt to cope with their troubles. They wanted it to go beyond dialogue about compliance with medication. This suggests that workers need to find a way to ‘reach through’ the psychotic beliefs, to engage the person meaningfully, to gain their trust, to reach past the threat and the authority that they represent as part of their role; processes described by Banks, Stroud and Doughty [63] as ‘personalisation’. Of relevance to this need, Suetani, Foo and Wilson’s [64] study involving 90 mental health patients found a trend for greater compliance to depot medication by those not on a CTO compared with those who were. They concluded that CTOs become a double-edged sword because, while forced cooperation might lead some patients to accept medication in the long-term, other patients might associate the experience with a loss of autonomy and develop negative attitudes to medication more generally. This might also include negative attitudes to help-seeking from mental health services and indeed the medical profession.

Several worker participants’ descriptions of their interactions with patients on CTOs appeared to focus on medication compliance as central to their practice. Several patient participants perceived this as the main agenda of workers’ interactions with them, at the exclusion of other forms of support. Stratford et al. [52] acknowledged this focus on medication as one of a number of challenges facing mental health care in Australia, impeding the growth of recovery-based approaches. They argue that this focus deskills workers and encourages coercion as a currency for engaging with and treating people with mental illness. Stratford et al. [52] conclude that the promotion of health professionals as the ‘experts’ disempowers patients, minimizing their role and contribution, and therefore stifling recovery that would otherwise promote patients’ control over their own lives.

Bracken et al. [65] argued that the non-technical aspects of interventions (such as the development of meaningful, non-judgmental relationships) are as much involved in recovery as the therapies and psychiatric medications used to treat mental illness. What they appear to be describing is a need for greater empathy and respect for clients’ experience [50]. Honneth’s [66] Recognition and Disrespect theory explores the moral experiences of disrespect. They outline three types of disrespect during which patients are not recognized as deserving the respect normally accorded to them: actual body is assaulted (eg with forced medications); rights taken away (eg. detention); and whole mode of living is culturally downgraded (pp.132–133). He calls for greater communicative and ethical competence by workers. For several of the patient and worker participants, the patients’ views were no longer seen as valid or truthful; as Vicky and Peter exemplified, ‘There was no second chance at trust’. This meant that a shared commitment to reaching agreement was difficult and workers were more likely to move to coercive practices.

### Limitations

As noted previously [47], this study involved a small sample of patients and workers. All worker participants were drawn from one mental health service in Australia. Therefore, results may not be generalizable to other jurisdictions. Other studies have noted bias in recruiting participants who might have more positive regard for CTOs. Our study did not have this limitation. The interviewer’s status as a consumer advocate may have assisted participants to speak more freely. The lead researcher was a clinician within mental health services between 1996 and 2007. She was aware of three of the patient participants, but she had not been directly involved in their care as a case manager during that time. The lead researcher was aware of six of the staff participants because of her prior clinical role within mental health services. She had not had any direct or regular contact with any of these participants since leaving mental health services in 2007. All participants were unknown to all other members of the research team. These conditions and shared analysis of data by the research team helped to minimise any bias in reporting.

The study did not include the views of those people on a CTO who met the exclusion criteria for this study. A further limitation was that the sample did not include patients at the earlier stage of their CTO or patients on the least restrictive order type (currently 28 days for South Australia). People at an earlier stage of their CTO or on shorter term CTOs might have had different perspectives to share, with different emphases to make about their experiences. The vast majority of CTOs currently applied for and imposed in South Australia are for a 12-month period [67]; hence, our sample likely reflects the dominant statutory process used within the community from where our participants were recruited.

## Conclusion

The administration of CTOs is complex because of inherent dilemmas associated with coercion. However, by acknowledging the role of metaphors in these patients’ lives, workers could enhance opportunities to engage these patients in a more meaningful dialogue about their personal experiences as an alternative to practice predominantly focused on risk. Such a dialogue could also enhance workers’ reflection on their work with people on CTOs. Understanding the CTO experience through an examination of the metaphors used by patients on CTOs and dialoguing with patients about those metaphors could offer opportunities for delivering more person-centred care to this population within this complex care situation. Its importance lies in its potential to assist workers in accessing the experiences of patients on CTOs, with implications for building trust and rapport, and offering a bridge to improve recovery-based practice. This research has shown that more understanding of how to promote autonomy, capacity and supported decision-making, and how to address the impacts of coercion, through workers' use of power and control inherent within the CTO process, is needed. Understanding and acknowledging the role of metaphors offers a way forward.

### Ethics approval and consent to participate

This study received ethical clearance from the SA Health Human Research Ethics Committee (HrEC/12/SAH/61). Permission to conduct the study was also granted by the relevant clinical and service directors for the mental health services involved. All participants were provided with a participant information sheet and provided their signed informed consent to participate in the study.

### Consent for publication

Not applicable. As part of ethics approval and consent, all participants also provided their consent for their de-identified interview data to reported in any publications arising from this research.

### Availability of data and materials

The raw qualitative data for this study is currently stored on a secure server, due to its sensitive nature. The de-identified data can be made available upon request to the corresponding author.

## Abbreviations

CDA: critical discourse analysis
CTO: community treatment order
ITO: involuntary treatment order
